# Therapeutic Potential of Santa Herba Extract in Obesity: Impact on Lipid Metabolism and Hormonal Balance

**DOI:** 10.1002/fsn3.70479

**Published:** 2025-06-17

**Authors:** Young‐Hee Jo, Eun‐Mi Hong, Sang‐Back Kim, Ju Gyeong Kim, Sung‐Jin Lee, Wansuk Son, Min‐Jung Ma, Sung Dae Kim, Joo‐Hee Choi, Min‐Soo Seo

**Affiliations:** ^1^ Food Science R&D Center KolmarBNH Co., Ltd. Seoul Republic of Korea; ^2^ Department of Nutraceutical Ingredients Research FINE BS Co., Ltd. Seoul Republic of Korea; ^3^ College of Veterinary Medicine, Kyungpook National University Daegu Republic of Korea; ^4^ Preclinical Research Center Daegu‐Gyeongbuk Medical Innovation Foundation Daegu Republic of Korea

**Keywords:** body weight, caloric intake, flavonoids, homoeriodictyol, lipid accumulation, metabolic regulation

## Abstract

Many studies have reported that flavonoids can effectively suppress metabolic diseases related to obesity. Santa Herba extract (SHE), which is rich in flavonoids, has shown potential anti‐obesity effects through clinical evaluations, but its anti‐obesity mechanisms remain unclear. Therefore, an obese mouse model was established to further investigate its underlying mechanisms and biological effects. C57BL/6 mice were fed a high‐fat diet (HFD) for 4 weeks to induce obesity and subsequently treated for 12 weeks with Orlistat (30 mg/kg) or SHE (50, 100, or 200 mg/kg). Body weight, food intake, fat mass (DEXA), serum biochemistry, histological changes, and gene/protein expression in liver and adipose tissue were analyzed. SHE200 reduced body weight by approximately 10%, fat mass by 15%, liver weight by nearly 40%, and epididymal adipocyte size by about 24% compared to the HFD group. Serum HDL was increased by approximately 1.2‐fold, while LDL, ALT, and AST levels were reduced to 0.8‐, 0.5‐, and 0.6‐fold of HFD levels, respectively. Leptin levels were also reduced to 0.6‐fold of HFD levels, reflecting improvements in hormonal balance. In adipose tissue, FAS and ACC were reduced to approximately 0.6‐fold of HFD levels, while key adipogenic transcription factors SREBP1c, CEBPα, and PPARγ were decreased to 0.5‐, 0.6‐, and 0.3‐fold, respectively. PGC1α and CPT1α expression were modulated by SHE treatment, showing a 1.9‐fold increase and 0.4‐fold reduction, respectively. In liver tissue, similar reductions were observed, with FAS and ACC downregulated to 0.6‐ and 0.7‐fold, and SREBP1c, CEBPα, and PPARγ suppressed to 0.4‐, 0.5‐, and 0.3‐fold, respectively. Notably, PGC1α expression increased by approximately 2.2‐fold, while CPT1α was reduced to about 0.5‐fold. The findings underscore the potential of SHE as a natural, multi‐targeted therapeutic agent for managing obesity and associated metabolic disorders.

## Graphical abstract

Santa Herba extract (SHE), a flavonoid‐rich natural compound, was evaluated for its anti‐obesity effects in a high‐fat diet (HFD)‐induced obesity model using C57BL/6 mice. SHE treatment significantly reduced body weight, adipocyte size, and liver lipid accumulation, while modulating key lipogenic and adipogenic gene expression. These findings highlight the potential of SHE as a natural therapeutic agent for obesity and metabolic disorders.

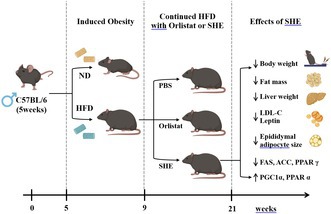

**Abbreviations**
A/L: adiponectin/leptin
ACC: acetyl‐CoA carboxylase
ALT: alanine aminotransferase
AST: aspartate aminotransferase
CEBPα: CCAAT/enhancer‐binding protein α
CPT1α: carnitine palmitoyltransferase 1 α
DEXA: dual‐energy X‐ray absorptiometry
FAS: fatty acid synthase
HDL: high‐density lipoprotein
H&E: hematoxylin and eosin
HFD: high‐fat diet
LDL: low‐density lipoprotein
ND: normal diet
PGC1α: peroxisome proliferator‐activated receptor gamma coactivator 1 α
PPARα: peroxisome proliferator‐activated receptor α
PPARγ: peroxisome proliferator‐activated receptor γ
SHE: Santa Herba extract
SREBP1c: sterol regulatory element‐binding protein 1 c
TC: total cholesterol

## 1 Introduction

Obesity is a complex metabolic disorder characterized by excessive fat accumulation resulting from an imbalance between caloric intake and energy expenditure (Lin and Li 2021). It typically stems from a calorie‐dense, nutritionally imbalanced diet, which leads to an increase in adipose tissue (Oussaada et al. 2019). In 2022, over 1 billion people globally were affected by obesity, more than double the number since 1990. Obesity rates among children and adolescents (ages 5–19) have quadrupled during this period (NCD‐RisC 2024). It is a chronic condition that significantly increases the risk of multiple comorbidities, including hypertension, insulin resistance, type 2 diabetes, coronary artery disease, stroke, and certain cancer types (Studentsova et al. 2018). Owing to the serious health risks associated with obesity, effective treatment strategies are essential. Because many individuals struggle to maintain long‐term weight loss through diet and exercise alone, pharmacological therapies have become crucial for effective obesity management (Aaseth et al. 2021). Approved anti‐obesity drugs (such as Orlistat, which inhibits fat absorption, and Liraglutide, a GLP‐1 receptor agonist) have shown efficacy in promoting weight loss (Leventhal‐Perek et al. 2023). However, these drugs are linked to various side effects, including gastrointestinal discomfort, increased heart rate, and insomnia (Cheung et al. 2013; Tak and Lee 2020). Consequently, ongoing research focuses on developing treatments that are safer for long‐term use and more effective for sustained weight management. Despite advancements, no pharmacological treatment has achieved long‐term weight reduction and health benefits comparable to those of bariatric surgery (Müller et al. 2022).

Given the limitations and side effects of current pharmacological treatments, interest in developing natural‐source anti‐obesity therapies is growing (Kazemipoor et al. 2015; Shaik Mohamed Sayed et al. 2023). Studies show that natural plant products, particularly those rich in bioactive compounds such as flavonoids and polyphenols, possess anti‐obesity properties. These compounds exert antioxidant, anti‐inflammatory, and lipid metabolism‐regulating effects, reducing oxidative stress and inflammation—key contributors to obesity‐related metabolic imbalance (Hossain et al. 2016; Jayarathne et al. 2017; Boccellino and D'Angelo 2020). Recent studies have reported that polyphenol‐rich herbal extracts, such as green tea polyherbal formulations, also demonstrate significant anti‐obesity potential through the modulation of lipid metabolism and inflammatory responses (Pandeya et al. 2021). These findings support the therapeutic potential of polyphenol‐based natural products in obesity management.

Flavonoids, including sterubin, eriodictyol, homoeriodictyol, hesperidin, and luteolin, are recognized for their role in supporting weight management and metabolic health (Jayarathne et al. 2017; Nani et al. 2021). Studies show that these compounds modulate lipid metabolism, inhibit fat cell differentiation, and enhance lipid breakdown, contributing to weight management and improved metabolic health (Oliveira et al. 2022; Ramírez‐Moreno et al. 2022).

Santa Herba, or Yerba Santa ( *Eriodictyon californicum* ), is particularly rich in polyphenols and flavonoids, which have been utilized as food supplements and therapeutic agents owing to their health benefits (Mödinger et al. 2021). Compounds in Santa Herba exhibit neuroprotective activity through antioxidant and anti‐inflammatory effects (Fischer et al. 2019; Hofmann et al. 2020; Mokdad‐Bzeouich et al. 2016). A recent clinical study further reveals that the antioxidant properties of Santa Herba extract (SHE) support weight and fat reduction in obese individuals by enhancing energy metabolism (Mödinger et al. 2021). Although clinical studies have demonstrated the promising anti‐obesity effects of SHE, the underlying molecular mechanisms remain unclear. Animal studies are necessary to investigate detailed pathways, such as lipid metabolism and adipogenesis, which cannot be fully explored in human subjects due to ethical and practical constraints. Therefore, this study aims to elucidate the mechanistic basis of the anti‐obesity effects of SHE using a high‐fat diet (HFD)‐induced obesity mouse model. Therefore, this study aims to investigate the potential anti‐obesity effects of SHE in a HFD‐induced obesity mouse model. We administered SHE to mice for 4 weeks while inducing obesity with a HFD to evaluate its effects on weight gain and metabolic health markers.

## 2 Materials and Methods

### 2.1 Preparation of the Santa Herba Extract (SHE)

The SHE powder (SantEnergy Nu) used in this study was commercially obtained from Mibelle AG (Buchs, Switzerland). According to the manufacturer, SHE was prepared by extracting the dry aerial parts of *Eriodictyon californicum* with 35% ethanol at 18°C for 1 h by percolation, followed by vacuum concentration and spray drying on 30% gum arabic as a carrier. The extract is standardized to contain at least 4.0% homoeriodictyol, based on ultra‐performance liquid chromatography (UPLC) analysis. For animal administration, the SHE powder was freshly dissolved in phosphate‐buffered saline (PBS) and orally administered to mice at the indicated doses.

### 2.2 Ultra‐Performance Liquid Chromatography Analysis

The flavonoid composition of the SHE was analyzed by the supplier, Mibelle AG (Buchs, Switzerland), using UPLC. The following is a summary of the analytical conditions. Extract analysis was performed using an ACQUITY C18 column (1.7 μm; 2.1 × 50 mm; Waters AG, Switzerland) connected to an UPLC system (Waters Acquity Classic; Waters AG, Switzerland). The run was performed using solvent A (H_2_O + 0.05 trifluoracetic acid) and solvent B (methanol) with a gradient of 30%–95% solvent B over 5 min with a flow rate of 0.40 mL/min followed by a wash‐out with 95% solvent B for 1 min. UV‐detection was performed at 288 nm using a UV detection system (Shimadzu, Kyoto, Japan). SHE (1 g) was dissolved in 10 mL of methanol, vortexed for 2 min, and filtered through a 0.22 μm syringe filter before injection. A 2 μL aliquot of the filtered solution was injected into the UPLC system for analysis. Reference substances homoeriodictyol, eriodictyol and sterubin (all received from Extrasynthese, Lyon, France) were used as standard for compound identification. The analytical approach is consistent with previously published methods (Baumann et al. 2025).

### 2.3 Animal Studies

All animal experimental protocols were reviewed and approved by the Institutional Animal Care and Use Committee (IACUC) of the Preclinical Research Center at KMEDIHUB (approval No. KMEDI‐23031301‐00, approved on March 3, 2023, Daegu, Korea) and all procedures adhered to the IACUC guidelines. Forty‐eight male C57BL/6 mice, aged 5 weeks, were purchased from Koatech (Seoul, Korea). After a 1‐week acclimation period, the mice were divided into two groups to receive a normal diet (ND, 10% kcal fat, D12450B; Research Diet, New Brunswick, NJ, USA) or HFD (60% kcal fat, D12492; Research Diet, New Brunswick, NJ, USA) for 16 weeks. The HFD was administered to induce obesity, which occurred after the initial 4 weeks. Following this, the mice were randomly assigned to the following six experimental groups: (1) ND, *n* = 8; (2) HFD, *n* = 8; (3) HFD with Orlistat (Tokyo Chemical Industry, Tokyo, Japan) (30 mg/kg), *n* = 8; (4) HFD with SHE (50 mg/kg), *n* = 8; (5) HFD with SHE (100 mg/kg), *n* = 8; and (6) HFD with SHE (200 mg/kg), *n* = 8. The dose of Orlistat (30 mg/kg) was selected based on a previously published study investigating its anti‐obesity effects in a HFD‐induced obese rodent model (Lee et al. 2019). From weeks 4 to 16, the mice were administered Orlistat or SHE once daily via oral gavage after preparation in PBS at the specified doses. Body weight and food intake were measured weekly throughout the experiment to assess the effects of the diet and treatments. At the end of 16 weeks, the mice were fasted for approximately 16 h overnight (Jung et al. 2024) and euthanized, and blood and tissue samples were collected for further analysis.

### 2.4 Body Composition Analysis Using DEXA

One day before euthanasia, dual‐energy X‐ray absorptiometry (DEXA) was conducted using a total‐body scanner (Osteosys; Insight) to assess body fat distribution and quantity. The mice were sedated with isoflurane through gas inhalation and positioned in a prone position on the scanning table for the DEXA scan. Each scan took approximately 25 s, including 10 s of radiation exposure. Body fat (g) values were calculated using the software of the manufacturer, which excluded bone areas from the X‐ray images.

### 2.5 Serum Biochemical Analysis

Blood samples were collected from the abdominal vena cava of the mice and centrifuged at 3000 rpm for 20 min to separate the serum, which was stored at −80°C for further analysis. Serum lipid profiles and liver function markers were measured with an automatic clinical chemistry analyzer (TBA‐120FR; Toshiba). The analytes included glucose, total cholesterol, triglycerides, high‐density lipoprotein (HDL), low‐density lipoprotein (LDL), alanine aminotransferase (ALT), and aspartate aminotransferase (AST). Levels of leptin and adiponectin were measured using Quantikine ELISA kits (MOB00B for leptin and MRP300 for adiponectin; R&D Systems, MN, USA) following the instructions of the manufacturer.

### 2.6 Histological Analysis

Epididymal adipose tissue and liver were fixed in 10% neutral‐buffered formalin, before being processed using a tissue processor (Thermo Fisher Scientific Inc., Runcorn, UK). The fixed tissues were embedded in paraffin, sectioned to 4 μm thickness, and mounted on glass slides. The sections were stained with hematoxylin and eosin (H&E) using an autostainer (Dako CoverStainer; Agilent, Santa Clara, CA, USA). The stained slides were examined under a microscope, and adipocyte sizes were measured.

### 2.7 mRNA Expression Analysis

Total RNA was extracted from the epididymal adipose tissue and liver of mice using TRIzol reagent. Briefly, the tissues were lysed with TRIzol, and chloroform was added to facilitate phase separation. The RNA‐containing aqueous phase was collected, and RNA was precipitated with isopropanol. The RNA pellet was then washed with ethanol, air‐dried, and dissolved in RNase‐free water. RNA quality and concentration were assessed using a NanoDrop spectrophotometer. For cDNA synthesis, 1 μg of isolated RNA was reverse transcribed using an RT premix (TOYOBO Biotechnology, Osaka, Japan) following the instructions of the manufacturer. Quantitative real‐time PCR (qPCR) was conducted using QGreen 2X SYBR Green PCR Master Mix (CellSafe, Yongin, Republic of Korea) and gene‐specific primers (Table 1).

**TABLE 1:** Primer (mouse) sequences for real time PCR.

Primer	Primer	Sequences
FAS	Forward	GGCCCCTCTGTTAATTGGCT
	Reverse	GGATCTCAGGGTTGGGGTTG
ACC	Forward	GGCAGCAGTTACACCACATAC
	Reverse	TCATTACCTCAATCTCAGCATAGC
SREBP1c	Forward	CGAGATGTGCGAACTGGACA
	Reverse	GGAGGCCAGAGAAGCAGAAG
CEBPα	Forward	GCAAAGCCAAGAAGTCGGTG
	Reverse	TCACTGGTCAACTCCAGCAC
PPARγ	Forward	TTGCTGTGGGGATGTCTCAC
	Reverse	AACAGCTTCTCCTTCTCGGC
PGC1α	Forward	CGGAAATCATATCCAACCAG
	Reverse	TGAGGACCGCTAGCAAGTTTG
CPT1α	Forward	AAGGAGGGAGACTTCCAACG
	Reverse	CTCCCACCAGTCACTCACAT
PPARα	Forward	AGACAAAGAGGCAGAGGTCC
	Reverse	AAGGAGGACAGCATCGTGAA
GAPDH	Forward	AACTTTGGCATTGTGGAAGG
	Reverse	ACACATTGGGGGTAGGAACA

### 2.8 Protein Expression Analysis

Protein extraction from epididymal adipose tissue and liver was conducted using RIPA buffer supplemented with a protein phosphatase inhibitor. Protein concentration was then assessed using the Pierce BCA Protein Assay Kit (Thermo Fisher Scientific, Waltham, MA, USA) according to the instructions of the manufacturer. Protein lysates were separated on a 10% SDS–PAGE gel and transferred onto nitrocellulose membranes. For Western blot analysis, 80 μg of adipose tissue lysate and 30 μg of liver tissue lysate were loaded per lane. The membranes were blocked with EveryBlot Blocking Buffer (Bio‐Rad, Hercules, CA, USA) for 1 h at room temperature, then incubated overnight at 4°C with primary antibodies targeting FAS and ACC (Cell Signaling Technology, Danvers, MA, USA) and β‐actin (Santa Cruz Biotechnology Inc., Santa Cruz, CA, USA). All primary antibodies were diluted at 1:5000. After incubation with secondary antibodies (diluted at 1:10,000), protein band intensity was detected using the ECL solution detection kit (EzWestLumi Pico, Dogen, Seoul, Korea) and analyzed with the ImageQuant LAS 4000 system (GE Healthcare, Buckinghamshire, UK).

### 2.9 Statistical Analysis

Calculations were performed with GraphPad Prism version 8.0.1 (GraphPad Software, San Diego, CA, USA). All data are presented as mean ± SD. Student's *t*‐test or one‐way ANOVA followed by Dunnett's post hoc test was used to calculate *p*‐values for single‐point comparisons, while two‐way ANOVA with Dunnett's post hoc test was performed to examine the effects of treatment and time on mouse body weight changes. Statistical significance was set at *p* < 0.05.

## 3 Results

### 3.1 Phytochemical Profile of SHE

**FIGURE 1:**
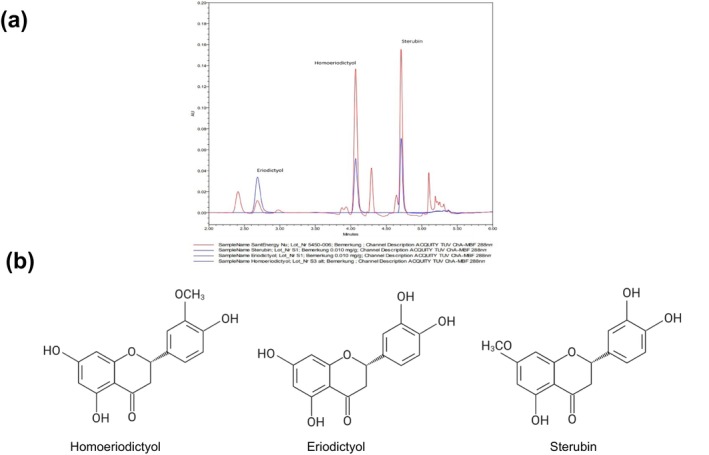
UPLC analysis of SHE components. (a) UPLC chromatogram of SHE (detection at 288 nm). The red line represents SHE, and the blue lines represent standards for homoeriodictyol, eriodictyol, and sterubin. (b) Chemical structure of homoeriodictyol, eriodictyol, and sterubin.

Santa Herba is known as a rich source of polyphenols, especially of various compounds of the flavonoid subgroup of flavanones, like eriodictyol, homoeriodictyol, sterubin, and hesperitin (Mödinger et al. 2021). A phytochemical analysis of the Santa Herba extract was performed using UPLC to elucidate its bioactive constituents. Our analysis based on UV‐detection at 288 nm revealed the presence of two prominent peaks corresponding to homoeriodictyol and sterubin (Figure 1a,b). Quantitative evaluation identified homoeriodictyol (55.1 mg/g) and sterubin (48.0 mg/g) as the dominant constituents, along with a smaller proportion of eriodictyol (6.9 mg/g). In addition to the individual flavonoid quantification, the total flavonoid content (TFC) of SHE was determined by the aluminum chloride colorimetric assay and measured at 187 mg/g. The total phenolic content (TPC) was determined using the Folin–Ciocalteu assay, resulting in 405 mg/g.

### 3.2 Effects of SHE on Body Weight Gain and Food Intake in HFD‐Fed Mice

**FIGURE 2:**
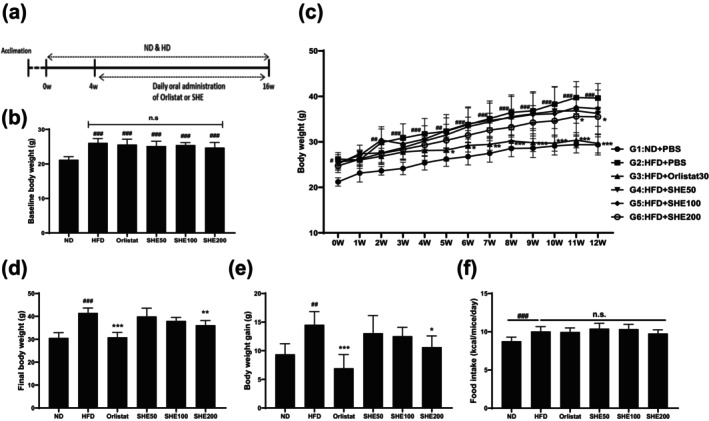
Experimental design and effects of SHE on body weight and food intake in HFD‐induced obesity model. (a) Experimental timeline schematic. Mice were acclimated for 1 week, then fed an HFD for 16 weeks. Oral administration of Orlistat or SHE began in week 5 and continued for 12 weeks. (b) Baseline body weight at the start of treatment (week 0; after 4 weeks of ND or HFD feeding). (c) Weekly body weight measurements throughout the study. Statistical significance: ^##^ *p* < 0.01, ^###^ *p* < 0.001 versus ND; *p < 0.05, **p < 0.01, ***p < 0.001 versus HFD using two‐way ANOVA (post hoc Dunnett's test). (d) Final body weight at study completion. (e) Total body weight gain. (f) Average daily food intake per mouse in each group. HFD, high‐fat diet; SHE, Santa Herba extract. Data are presented as mean ± SD (*n* = 8 per group). Statistical significance: ^##^ *p* < 0.01, ^###^ *p* < 0.001 versus ND; *p < 0.05, **p < 0.01, ***p < 0.001 versus HFD using one‐way ANOVA (post hoc Dunnett's test).

The experiment involved feeding mice with an ND or HFD for 16 weeks. After 4 weeks of obesity induction, daily oral administration of Orlistat or SHE was introduced as part of the treatment regimen (Figure 2a). At the start of treatment (week 0), body weight was significantly higher in the HFD group (25.9 ± 1.16 g, *p* < 0.001) compared to the ND group (21.2 ± 0.9 g), confirming the successful induction of obesity (Figure 2b). No significant differences were observed among the HFD‐fed groups at this baseline point. During the treatment period, body weight progressively increased in the HFD group (39.6 ± 3.18, *p* < 0.001) compared to that in the ND group (29.37 ± 2.24), indicating the successful establishment of the obesity model (Figure 2c). Orlistat (29.65 ± 2.09, *p* < 0.001), a known weight‐loss treatment, reduced body weight, bringing it closer to that of the ND group (Figure 2d,e). The SHE200 group (35.51 ± 2.08, *p* < 0.01) showed a significant reduction in body weight gain compared to that in the HFD group, indicating its potential anti‐obesity effects (Figure 2d,e). Caloric intake was significantly higher in the HFD group (10.04 ± 0.62, *p* < 0.001) than in the ND group (8.76 ± 0.52), with no significant differences observed among the HFD‐fed groups (Figure 2f).

### 3.3 Effects of SHE on Adipose Tissue Accumulation Indicators in HFD‐Fed Mice

**FIGURE 3:**
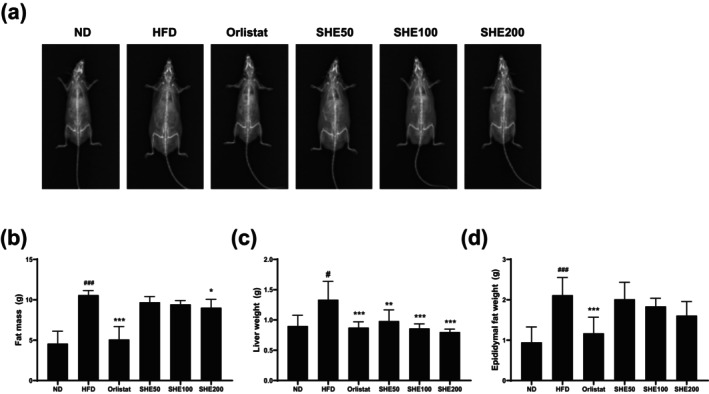
Effects of SHE on fat mass, liver weight, and epididymal fat weight in HFD‐induced obese mice. (a) Representative body composition images obtained through DEXA. (b) Total fat mass measured via DEXA. (c) Liver weight. (d) Epididymal fat weight. HFD, high‐fat diet; SHE, Santa Herba extract. Data are presented as mean ± SD (*n* = 8 per group). Statistical significance: ^#^ *p* < 0.05, ^###^ *p* < 0.001 versus ND; *p < 0.05, **p < 0.01, ***p < 0.001 versus HFD using one‐way ANOVA (post hoc Dunnett's test).

To evaluate the anti‐obesity effects of SHE, fat mass distribution in mice was assessed using DEXA. The analysis revealed that the HFD group (10.59 ± 0.55, *p* < 0.001) had significantly higher fat mass than that of the ND group (4.58 ± 1.52), while Orlistat treatment (5.08 ± 1.61, *p* < 0.001) effectively reduced fat accumulation (Figure 3a,b). SHE administration reduced fat accumulation compared to the HFD group, with a statistically significant reduction observed in the SHE200 group (9.03 ± 1.01, *p* < 0.05) (Figure 3a,b). Liver weight was significantly higher in the HFD group (1.34 ± 0.3, *p* < 0.05) than in the ND group (0.9 ± 0.17), but it decreased significantly following treatment with Orlistat (0.88 ± 0.09, *p* < 0.001) and SHE200 (0.8 ± 0.04, *p* < 0.01) (Figure 3c). Additionally, epididymal fat weight was significantly higher in the HFD group (2.12 ± 0.4, *p* < 0.001). Orlistat (1.17 ± 0.4, *p* < 0.001) significantly reduced epididymal fat weight, whereas SHE treatment showed a tendency to reduce it, although without statistical significance (Figure 3d).

### 3.4 Effects of SHE on Serum Profiles in HFD‐Fed Mice

**FIGURE 4:**
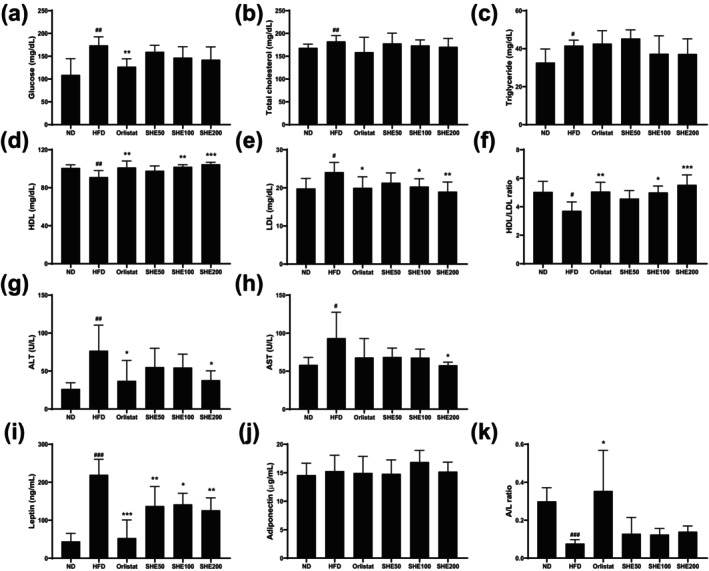
Effects of SHE on serum biochemical markers in HFD‐induced obese mice. (a) Serum glucose levels. (b) TC levels. (c) TG levels. (d) HDL levels. (e) LDL levels. (f) HDL/LDL ratio. (g) ALT levels. (h) AST levels. (i) Leptin levels. (j) Adiponectin levels. (k) A/L ratio. A/L, adiponectin/leptin; ALT, alanine aminotransferase; AST, aspartate aminotransferase; HDL, high‐density lipoprotein; HFD, high‐fat diet; LDL, low‐density lipoprotein; SHE, Santa Herba extract; TC, total cholesterol; TG, triglyceride. Data are presented as mean ± SD (*n* = 8 per group). Statistical significance: ^#^ *p* < 0.05, ^##^ *p* < 0.01, ^###^ *p* < 0.001 versus ND; *p < 0.05, **p < 0.01, ***p < 0.001 versus HFD using one‐way ANOVA (post hoc Dunnett's test).

HFD feeding (173.8 ± 18.7, *p* < 0.01) significantly increased serum glucose levels compared to the ND group (109 ± 35.7). While Orlistat treatment (127 ± 17.5, *p* < 0.01) significantly reduced glucose levels, SHE200 (138.8 ± 29.7) treatment showed a reduction trend compared to the HFD group, although this did not reach statistical significance (Figure 4a). No significant differences were observed in total cholesterol and triglyceride levels among the HFD‐fed groups (Figure 4b,c). HDL‐cholesterol levels, which were reduced by HFD feeding (91.16 ± 6.96, *p* < 0.01), were elevated with Orlistat (101.3 ± 6.74, *p* < 0.01) and SHE treatment at 100 (102 ± 2.2, *p* < 0.01) and 200 (104.7 ± 2.1, *p* < 0.001) mg doses (Figure 4d). Conversely, LDL‐cholesterol levels were higher in the HFD group (24.1 ± 2.54, *p* < 0.05) than in the ND group (19.8 ± 2.63, *p* < 0.05), but this increase was reduced by Orlistat (20 ± 2.8, *p* < 0.05) and SHE100 (20.3 ± 2.06, *p* < 0.05) and SHE200 (19 ± 2.52, *p* < 0.01) treatments (Figure 4e). The HDL/LDL ratio was lower in the HFD group (3.71 ± 0.62, *p* < 0.05), while treatment with Orlistat (5.06 ± 0.65, *p* < 0.01) and SHE 100 (4.99 ± 0.45, *p* < 0.05) and 200 (5.53 ± 0.69, *p* < 0.001) elevated this ratio, suggesting an improvement in the lipid profile (Figure 4f). Serum liver enzyme levels were also assessed. HFD feeding (76.8 ± 33.6, *p* < 0.01) significantly elevated serum ALT levels, while treatment with Orlistat (37.2 ± 26.7, *p* < 0.05) and SHE200 (38 ± 12.2, *p* < 0.05) reduced these levels compared to that in the HFD group (Figure 4g). Similarly, serum AST levels were increased in the HFD group (93.5 ± 34.08, *p* < 0.05), with a significant reduction observed exclusively in the SHE200 group (57.8 ± 4.02, *p* < 0.05) (Figure 4h). Leptin and adiponectin levels were measured to assess the hormonal regulation of obesity and metabolic balance associated with HFD feeding. Leptin, which was elevated by HFD feeding (219.85 ± 40.84, *p* < 0.001) compared to the ND group (44.52 ± 21.27), was significantly reduced by Orlistat treatment (53.38 ± 47.7, *p* < 0.001). Furthermore, leptin levels were also significantly reduced in all SHE‐treated groups compared to the HFD group, although a clear dose‐dependent trend was not observed (SHE200: 126.38 ± 32.84, *p* < 0.01) (Figure 4i). No significant differences were observed in adiponectin levels among the groups (Figure 4j). The adiponectin/leptin (A/L) ratio was significantly increased in the Orlistat group (0.35 ± 0.21, *p* < 0.05) compared to the HFD group (0.07 ± 0.02, *p* < 0.001), whereas no significant changes were observed in the SHE‐treated groups (Figure 4k).

### 3.5 Effects of SHE on the Gene and Protein Expression Related to Lipogenesis, Adipogenesis, and Thermogenesis in WAT of HFD‐Fed Mice

**FIGURE 5:**
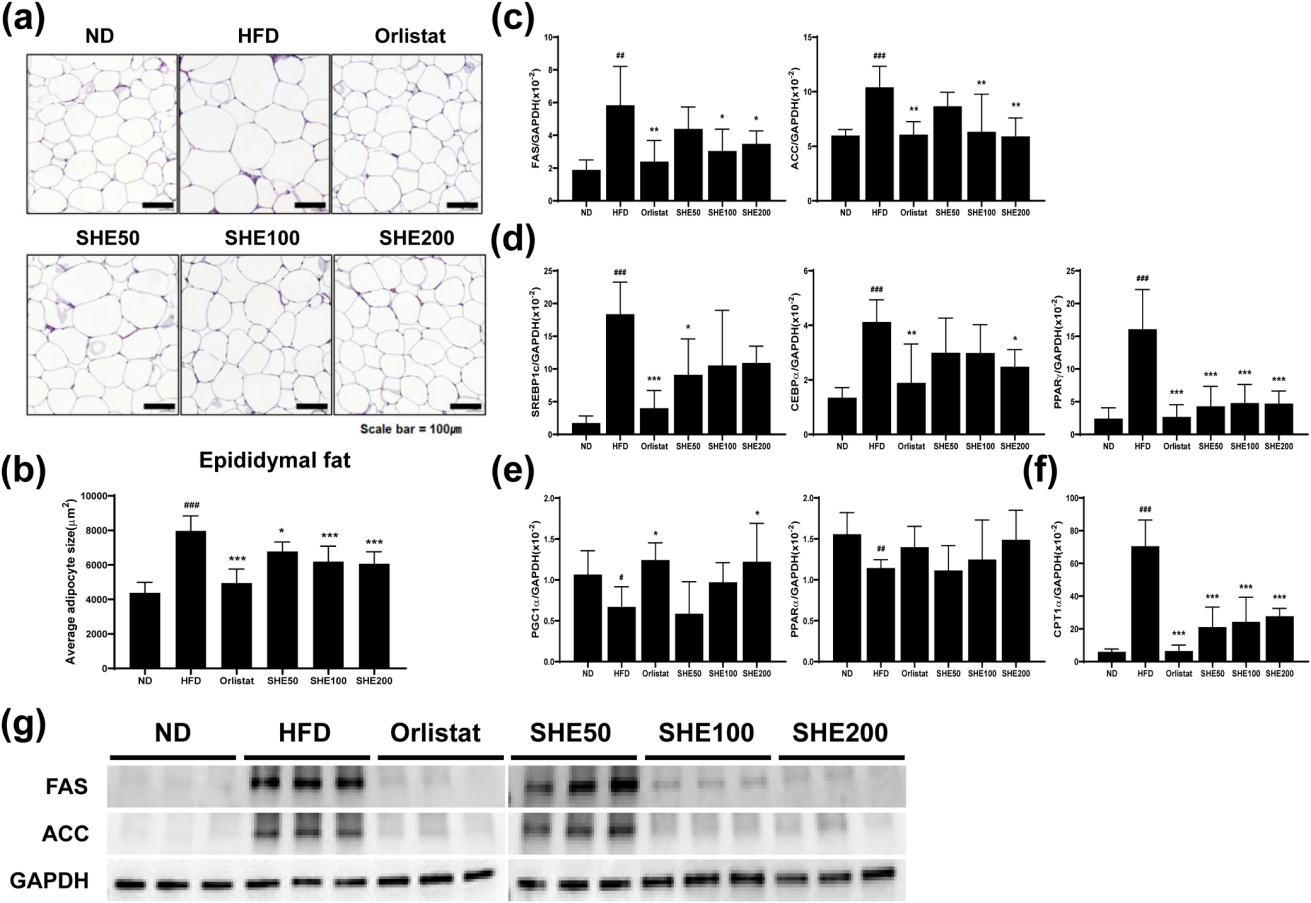
Effects of SHE on adipocyte size and gene expression related to lipogenesis, adipogenesis, and energy metabolism in epididymal adipose tissue of HFD‐induced obese mice. (a) Representative histological images of epididymal adipose tissue stained with H&E. Scale bar = 100 μm. (b) Average adipocyte size. (c) mRNA expression of lipogenic genes: FAS and ACC. (d) mRNA expression of adipogenic transcription factors: SREBP1c, CEBPα, and PPARγ. (e) mRNA expression of energy metabolism regulators: PGC1α and PPARα. (f) mRNA expression of the fatty acid oxidation regulator: CPT1α. (g) Protein expression of FAS and ACC as determined using Western blot analysis. HFD, high‐fat diet; SHE, Santa Herba extract. Data are presented as mean ± SD (*n* = 8 per group). Statistical significance: ^#^ *p* < 0.05, ^##^ *p* < 0.01, ^###^ *p* < 0.001 versus ND; *p < 0.05, **p < 0.01, ***p < 0.001 versus HFD using one‐way ANOVA (post hoc Dunnett's test).

To elucidate the anti‐obesity mechanisms of SHE, gene and protein expression associated with lipogenesis, adipogenesis, and thermogenesis in white adipose tissue were analyzed. Adipocyte size was measured using H&E‐stained sections of epididymal fat tissue. In the HFD group (7967.9 ± 870, *p* < 0.001), adipocytes in epididymal adipose tissue were significantly larger than those in the ND group (4378.44 ± 615). Treatment with Orlistat (4948.7 ± 811, *p* < 0.001) and SHE (SHE200: 6061 ± 699, *p* < 0.001) inhibited this increase in adipocyte size (Figure 5a,b). Figure 5c displays the expression levels of key lipogenic markers, including FAS and ACC. The HFD group (FAS: 5.83 ± 2.37, *p* < 0.01; ACC: 10.39 ± 1.92, *p* < 0.001) exhibited elevated expression of these markers compared to that of the ND group, indicating increased lipogenesis. However, Orlistat and SHE treatments significantly downregulated FAS and ACC expression, especially at higher doses. FAS expression was significantly reduced by Orlistat (2.39 ± 1.29, *p* < 0.01) and SHE200 (3.47 ± 0.79, *p* < 0.05), and ACC expression was similarly decreased by Orlistat (6.05 ± 1.19, *p* < 0.01) and SHE200 (5.89 ± 1.68, *p* < 0.01), suggesting reduced lipogenesis (Figure 5c). Adipogenic transcription factors SREBP1c, CEBPα, and PPARγ were significantly upregulated in the HFD group (SREBP1c: 18.36 ± 4.89, *p* < 0.001; CEBPα: 4.12 ± 0.81, *p* < 0.01; PPARγ: 16.06 ± 6.07, *p* < 0.001), indicating enhanced adipogenesis. Orlistat treatment significantly reduced the expression of all three genes (SREBP1c: 4.01 ± 2.7, *p* < 0.001; CEBPα: 1.88 ± 1.42, *p* < 0.01; PPARγ: 2.67 ± 1.85, *p* < 0.001). Among the SHE‐treated groups, SREBP1c expression was significantly reduced at SHE50 (9.1 ± 5.47, *p* < 0.05), CEBPα at SHE200 (2.48 ± 0.63, *p* < 0.05), and PPARγ was consistently downregulated across all SHE groups (SHE200: 4.7 ± 1.9, *p* < 0.001), suggesting an inhibitory effect of SHE on adipogenesis through dose‐ and gene‐specific mechanisms (Figure 5d). Thermogenic genes, including PGC1α and PPARα, were downregulated in the HFD group (PGC1α: 0.66 ± 0.24, *p* < 0.05; PPARα: 1.14 ± 0.1, *p* < 0.01) compared to the ND group. PGC1α expression was significantly increased by Orlistat (1.24 ± 0.21, *p* < 0.05) and SHE200 treatments (1.22 ± 0.47, *p* < 0.05). Although no statistical significance was observed for PPARα, its expression was increased in the Orlistat and SHE200 groups (1.48 ± 0.36) compared to the HFD group (Figure 5e). CPT1α, a key regulator of lipid metabolism, was upregulated in the HFD group (70.48 ± 16.04, *p* < 0.001) and reduced following treatment with Orlistat (6.46 ± 3.65, *p* < 0.001) and SHE treatment (SHE200: 27.7 ± 4.82, *p* < 0.001), suggesting a regulatory effect on lipid metabolism (Figure 5f). Western blot analysis confirmed that FAS and ACC protein levels were elevated in the HFD group but significantly reduced in the Orlistat‐ and SHE‐treated groups, particularly at higher SHE doses, supporting the observed suppression of lipogenesis induced by SHE (Figure 5g).

### 3.6 Effects of SHE on the Gene and Protein Expression Related to Lipogenesis, Adipogenesis, and Thermogenesis in Liver Tissue of HFD‐Fed Mice

**FIGURE 6:**
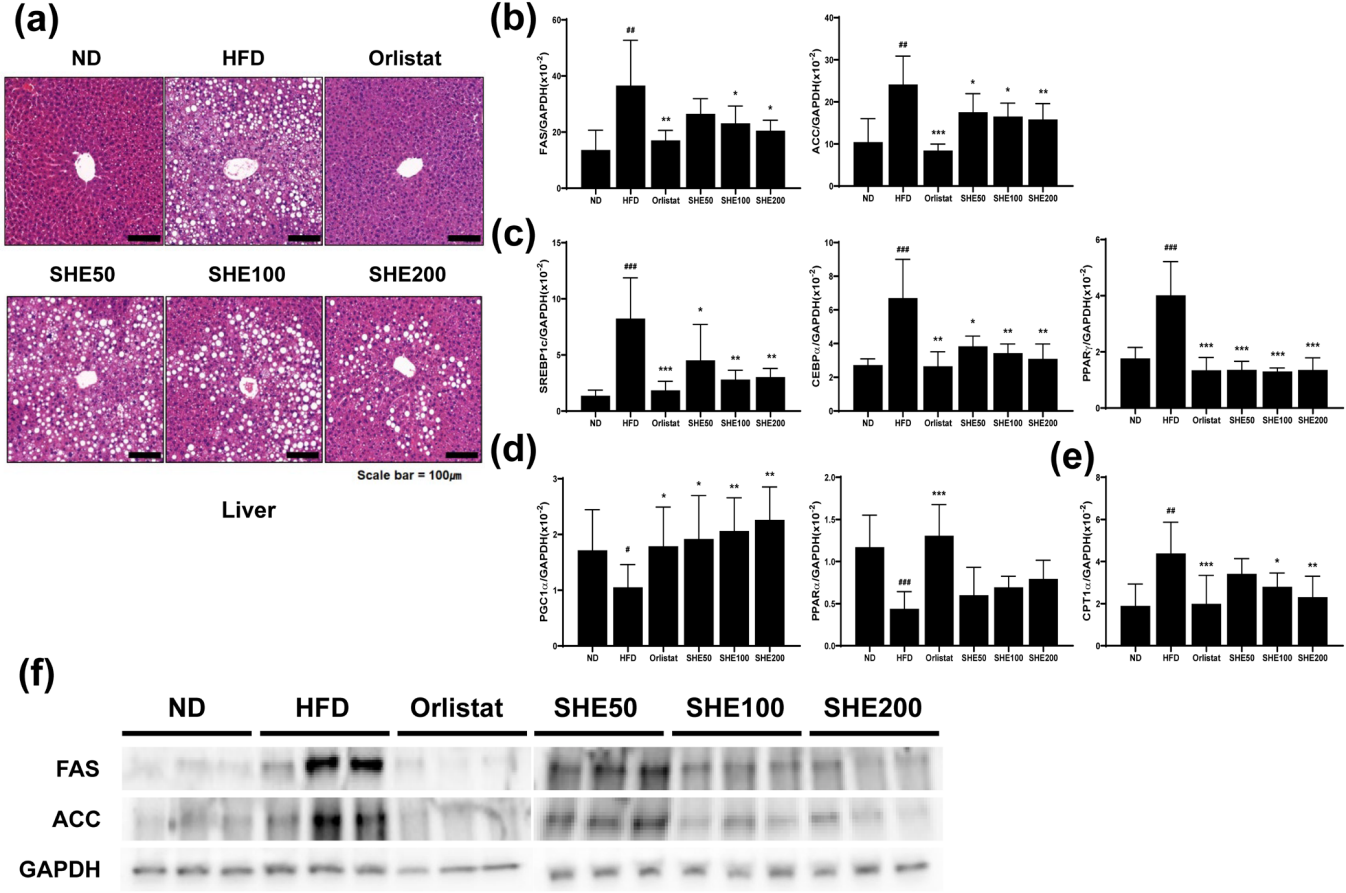
Effects of SHE on lipid accumulation and gene expression related to lipogenesis, adipogenesis, and energy metabolism in liver tissue of HFD‐induced obese mice. Representative histological images of liver tissue stained with H&E. Scale bar = 100 μm. (b) mRNA expression of lipogenic genes: FAS and ACC. (c) mRNA expression of adipogenic transcription factors: SREBP1c, CEBPα, and PPARγ. (d) mRNA expression of energy metabolism regulators: PGC1α and PPARα. (e) mRNA expression of the fatty acid oxidation regulator: CPT1α. (f) Protein expression of FAS and ACC as determined through Western blot analysis. HFD, high‐fat diet; SHE, Santa Herba extract. Data are presented as mean ± SD (*n* = 8 per group). Statistical significance: ^#^ *p* < 0.05, ^##^ *p* < 0.01, ^###^ *p* < 0.001 versus ND; *p < 0.05, **p < 0.01, ***p < 0.001 versus HFD using one‐way ANOVA (post hoc Dunnett's test).

To investigate the anti‐obesity effects of SHE in liver tissue, we analyzed histological changes and the expression of genes and proteins involved in lipogenesis, adipogenesis, and thermogenesis. Histological analysis with H&E staining revealed marked lipid accumulation in the liver tissue of the HFD group compared to that of the ND group, indicating hepatic steatosis. Treatment with Orlistat and SHE, particularly at higher doses, visibly reduced lipid droplet accumulation, indicating an ameliorative effect on hepatic lipid storage (Figure 6a). Lipogenic markers, including FAS and ACC, were significantly upregulated in the HFD group (FAS: 36.55 ± 16.16, *p* < 0.01; ACC: 24.13 ± 6.73, *p* < 0.01), reflecting increased lipogenic activity. Conversely, treatment with Orlistat (FAS: 17.04 ± 3.55, *p* < 0.01; ACC: 8.45 ± 1.52, *p* < 0.001) and SHE200 (FAS: 20.52 ± 3.69, *p* < 0.05; ACC: 15.81 ± 3.79, *p* < 0.01) significantly downregulated FAS and ACC expression, indicating a reduction in hepatic lipogenesis (Figure 6b). For adipogenesis, transcription factors such as SREBP1c, CEBPα and PPARγ were elevated in the HFD group (SREBP1c: 8.22 ± 3.63, *p* < 0.001; CEBPα: 6.69 ± 2.3, *p* < 0.001; PPARγ: 4.01 ± 1.2, *p* < 0.001). However, their expression was significantly reduced following treatment with Orlistat (SREBP1c: 1.83 ± 0.81, *p* < 0.001; CEBPα: 2.65 ± 0.86, *p* < 0.01; PPARγ: 1.34 ± 0.45, *p* < 0.001) and SHE (SHE200, SREBP1c: 3.02 ± 0.76, *p* < 0.01; CEBPα: 3.09 ± 0.88, *p* < 0.01; PPARγ: 1.35 ± 0.43, *p* < 0.001), with the strongest effects observed at higher SHE doses, indicating suppression of adipogenic pathways in liver tissue (Figure 6c). Thermogenic gene expression, including PGC1α and PPARα, was also evaluated in liver tissue. PGC1α expression, which was reduced by HFD feeding (1.05 ± 0.4, *p* < 0.05), was significantly increased by Orlistat treatment (1.78 ± 0.7, *p* < 0.05) and further enhanced by SHE200 (2.26 ± 0.59, *p* < 0.01), showing the highest expression among the groups. In contrast, PPARα expression showed no statistically significant change following SHE treatment, but a tendency toward increased expression was observed, similar to the pattern seen in adipose tissue (Figure 6d). Additionally, CPT1α expression was significantly upregulated in the HFD group (4.38 ± 1.48, *p* < 0.05) and reduced after Orlistat (1.98 ± 1.35, *p* < 0.001) and SHE treatment (SHE200: 2.3 ± 1, *p* < 0.01), indicating its regulatory effect on hepatic lipid metabolism (Figure 6e). Protein expression levels for FAS and ACC, assessed through Western blot, were elevated in the HFD group but significantly reduced following treatment with Orlistat and SHE, further supporting the observed suppression of lipogenesis induced by SHE (Figure 6f).

## 4 Discussion

This study investigates the anti‐obesity effects of SHE administered orally to C57BL/6 mice for 12 weeks, following an initial 4‐week period of HFD consumption. The results showed that the HFD significantly increased body weight, total fat mass, liver weight, and epididymal fat weight compared to that of the ND group. This finding is consistent with previous research findings, indicating that HFDs, owing to their high caloric density, promote excessive fat accumulation, resulting in weight gain and lipid deposition in the liver and adipose tissues (Li et al. 2020; Pemmari et al. 2022). The present study is the first to demonstrate the anti‐obesity effects of SHE in an animal model, expanding on prior clinical observations. SHE exerts multi‐faceted effects, including decreasing body weight, modulation of lipid metabolism, and suppression of adipogenesis. The identification of flavonoids such as homoeriodictyol and sterubin as major active compounds provides a novel mechanistic insight into its therapeutic potential.

SHE administration following the initial HFD period resulted in a dose‐dependent reduction in body weight and fat accumulation without altering overall food intake, suggesting that the anti‐obesity effects of SHE are likely due to modulating lipid metabolism and inhibiting fat storage rather than reducing caloric intake (Hossain et al. 2016). The reduction in liver weight and epididymal fat weight observed in SHE‐treated groups further supports its role in decreasing lipid accumulation in specific tissues. These findings align with findings from previous clinical studies, showing that SHE provides antioxidative and metabolic benefits, leading to reductions in body weight and body fat in overweight and obese individuals (Boccellino and D'Angelo 2020; Mödinger et al. 2021). These anti‐obesity effects of SHE can be attributed to its bioactive components. Our UPLC analysis of the SHE extract revealed that homoeriodictyol and sterubin were the predominant flavanones. Most probably, the major flavanones of SHE, particularly homoeriodictyol and sterubin, will contribute to the demonstrated anti‐obesity effects (Lin et al. 2025). Indeed, previous studies have reported that homoeriodictyol and sterubin exert anti‐inflammatory and metabolic regulatory activities, including suppression of adipogenesis and modulation of energy metabolism, which are closely associated with obesity management (Mödinger et al. 2021; Zhang et al. 2019). They also show chemical similarity to the flavonoids present in citrus extracts, which have been frequently investigated for their slimming effects (Im et al. 2024; Li et al. 2024). Additionally, these components may regulate lipid metabolism by promoting lipolysis and inhibiting adipogenesis, resulting in reduced fat deposition in the liver and adipose tissue (Chang et al. 2016; Fan et al. 2022).

Similarly, Orlistat effectively reduced body weight and fat accumulation in this study, aligning with its well‐known mechanism of inhibiting dietary fat absorption and promoting weight loss (Zakaria et al. 2021).

Obesity affects multiple metabolic markers, with glucose, total cholesterol, triglycerides, HDL, and LDL serving as key indicators of obesity‐related health status. HFD alters these markers, thereby increasing the risk of obesity‐related metabolic disorders (Steinberg and Carling 2019). Glucose levels were significantly lower in the Orlistat and SHE 200 treatment groups than in the HFD group, suggesting that Orlistat and SHE may reduce insulin resistance and improve glucose metabolism. This finding is consistent with that of previous studies indicating that these interventions enhance insulin sensitivity (Mittendorfer et al. 2001). Although total cholesterol and triglyceride levels were elevated in the HFD group, Orlistat and SHE treatment had no significant effect on these levels. Orlistat primarily reduces fat absorption, with a limited effect on certain plasma lipid levels (Filippatos et al. 2008). Thus, while SHE showed weight‐reducing effects, it did not exhibit a clear inhibitory effect on total cholesterol (TC) and TG levels. This observation may be attributed to tissue‐specific actions of SHE, such as suppression of adipogenesis or modulation of hepatic lipid metabolism, rather than systemic hypolipidemic mechanisms. Similar outcomes have been reported in previous studies where plant‐derived treatments led to reductions in body weight, adipocyte size, and hepatic steatosis, yet failed to produce significant changes in serum TC or TG levels (Kazemian et al. 2015; Yan et al. 2023). These results suggest that improvements in lipid handling may occur locally within metabolic tissues without necessarily translating to measurable changes in circulating lipid concentrations.

HDL levels modestly improved in the treatment groups, indicating a potential cardiovascular benefit, as HDL transports cholesterol from the bloodstream. In contrast, LDL levels were lower in the Orlistat and SHE groups, further supporting their potential role in reducing cardiovascular risks in HFD‐induced obese models (Wali et al. 2020). In this study, elevated ALT and AST levels in the HFD group indicate hepatic stress and potential liver damage due to an HFD (Toita et al. 2017). The reduction in liver enzyme levels with SHE treatment suggests its potential to prevent liver damage by reducing fat accumulation.

Elevated leptin levels in the HFD group reflect typical leptin resistance associated with obesity, where increased fat accumulation enhances leptin production while decreasing leptin sensitivity (Oda et al. 2008). The significant reduction in leptin levels in the SHE‐treated groups, particularly at 100 and 200 mg/kg, may reflect decreased fat accumulation owing to SHE administration. This finding aligns with results from a clinical trial in individuals with obesity, where SHE intake led to reduced leptin levels compared to that of placebo (Mödinger et al. 2021). In that trial, baseline leptin levels were higher in obese subjects, indicating leptin resistance and an imbalance in hunger–satiety regulation. The reduction in leptin with SHE suggests improved hormonal regulation. The consistent findings from animal and human studies underscore the potential of SHE to modulate leptin levels and promote metabolic balance, positioning it as a promising natural option for obesity management. Although adiponectin levels remained unchanged significantly, the improved A/L ratio in SHE‐treated groups suggests a healthier metabolic profile, potentially reducing insulin resistance and supporting metabolic balance (Castela et al. 2023). These findings suggest that SHE may serve as an effective natural alternative for reducing fat accumulation and managing obesity‐related hormonal imbalances.

Our findings show that SHE administration reduced adipocyte size in adipose tissue and decreased lipid droplet formation in liver tissue in a dose‐dependent manner following HFD induction. We hypothesized that SHE influences genes involved in lipogenesis, adipogenesis, and thermogenesis. ACC and FAS are critical enzymes in fatty acid synthesis. ACC converts acetyl‐CoA to malonyl‐CoA, initiating fatty acid synthesis, while FAS elongates fatty acid chains, leading to increased fat storage in adipocytes (Puljak et al. 2008; Ren et al. 2018). The transcription factors SREBP‐1c, C/EBPα, and PPARγ regulate glucose and energy metabolism, as well as lipid synthesis and metabolism (Cho et al. 2022; Ferré et al. 2021; Yang and Shang 2023). Following SHE treatment, the expression of these genes decreased, similar to the effects observed with Orlistat administration, suggesting that SHE may regulate lipid metabolism, thereby reducing fat accumulation in adipose and liver tissues. Consequently, SHE has the potential as a natural agent for modulating lipid metabolism and preventing diet‐induced fat accumulation.

Moreover, SHE treatment modulates energy metabolism regulators. PGC‐1α and PPARα, which were reduced by the HFD, responded differently to the treatments. PGC‐1α expression was significantly restored by Orlistat and SHE treatment, indicating enhanced mitochondrial function and energy expenditure (Besseiche et al. 2015). In contrast, although PPARα did not show a statistically significant increase following SHE treatment, a tendency toward upregulation was observed, particularly in the SHE200 group. Given that PPARα activates genes involved in fatty acid transport and oxidation, which are crucial for lipid metabolism in high‐energy‐demand tissues (Yang et al. 2015), this trend suggests a potential positive effect of SHE on energy metabolism, although further validation is needed.

Here, elevated CPT1 levels in the HFD group indicate a compensatory response to excess fatty acids, as CPT1 is essential for mitochondrial fatty acid oxidation (Lee et al. 2015). However, elevated CPT1 levels may also induce metabolic stress and inflammation owing to excessive lipid oxidation (Han et al. 2015). Treatment with SHE and Orlistat reduces CPT1 expression, likely by decreasing fat accumulation and the need for compensatory oxidation. This downregulation correlates with reduced adipocyte size and liver lipid content, suggesting that SHE and Orlistat restore metabolic balance by modulating lipid metabolism (Fujiwara et al. 2018). Despite its promising findings, this study has several limitations. First, only male mice were used, which may not fully capture the gender‐specific metabolic responses to SHE. Second, the study duration may have been insufficient to assess long‐term effects on cholesterol and triglyceride levels, which remained unchanged despite reductions in body weight. Further studies should investigate these aspects and include multi‐organ assessments to better understand SHE's systemic effects.

## 5 Conclusions

This study reveals that SHE effectively reduces body weight, adipose tissue size, and liver lipid accumulation in an HFD‐induced obesity model. These effects stem from its modulation of key metabolic pathways involved in lipid synthesis, adipogenesis, and energy metabolism, including decreased lipogenic gene expression and restored energy metabolism regulators. Additionally, SHE lowers leptin levels and improves the adiponectin‐to‐leptin ratio, suggesting enhanced hormonal balance and metabolic health. These findings highlight the potential of SHE as a natural agent for managing obesity and related metabolic disorders, presenting it as a promising treatment option.

## Author Contributions

**Young‐Hee Jo:** conceptualization (equal), data curation (equal). **Eun‐Mi Hong:** conceptualization (equal), data curation (equal), resources (equal). **Sang‐Back Kim:** data curation (equal), formal analysis (equal). **Ju Gyeong Kim:** data curation (equal), formal analysis (equal). **Sung‐Jin Lee:** data curation (equal), formal analysis (equal). **Wansuk Son:** investigation (equal), methodology (equal). **Min‐Jung Ma:** investigation (equal), methodology (equal). **Sung Dae Kim:** investigation (equal), methodology (equal). **Joo‐Hee Choi:** investigation (equal), visualization (equal), writing – original draft (equal). **Min‐Soo Seo:** supervision (equal), writing – original draft (equal), writing – review and editing (equal).

## Conflicts of Interest

The authors declare no conflicts of interest.

## Data Availability

The datasets used and/or analyzed during the current study are available from the corresponding author on reasonable request.

## References

[fsn370479-bib-0001] Aaseth, J. , S. Ellefsen , U. Alehagen , T. M. Sundfør , and J. Alexander . 2021. “Diets and Drugs for Weight Loss and Health in Obesity – An Update.” Biomedicine & Pharmacotherapy 140: 111789. 10.1016/j.biopha.2021.111789. 34082399

[fsn370479-bib-0002] Baumann, J. , E. Bönzli , F. Wandrey , and T. Grothe . 2025. “Moldavian Dragonhead Extract: A Natural Collagen‐Booster to Target Skin Aging.” OBM Geriatrics 9, no. 2: 1–22. 10.21926/obm.geriatr.2502305.

[fsn370479-bib-0003] Besseiche, A. , J. Riveline , J. Gautier , B. Bréant , and B. Blondeau . 2015. “Metabolic Roles of PGC‐1α and Its Implications for Type 2 Diabetes.” Diabetes & Metabolism 41, no. 5: 347–357. 10.1016/j.diabet.2015.02.002. 25753246

[fsn370479-bib-0004] Boccellino, M. , and S. D'Angelo . 2020. “Anti‐Obesity Effects of Polyphenol Intake: Current Status and Future Possibilities.” International Journal of Molecular Sciences 21, no. 16: 5642. 10.3390/ijms21165642. 32781724 PMC7460589

[fsn370479-bib-0005] Castela, I. , J. Morais , I. BarreirosMota , et al. 2023. “Decreased Adiponectin/Leptin Ratio Relates to Insulin Resistance in Adults With Obesity.” American Journal of Physiology. Endocrinology and Metabolism 324, no. 2: E115–E119. 10.1152/ajpendo.00273.2022. 36351292

[fsn370479-bib-0006] Chang, Y. , M. Yang , S. Chen , and C. Wang . 2016. “Mulberry Leaf Polyphenol Extract Improves Obesity by Inducing Adipocyte Apoptosis and Inhibiting Preadipocyte Differentiation and Hepatic Lipogenesis.” Journal of Functional Foods 21: 249–262. 10.1016/j.jff.2015.11.033.

[fsn370479-bib-0007] Cheung, B. , T. Cheung , and N. Samaranayake . 2013. “Safety of Antiobesity Drugs.” Therapeutic Advances in Drug Safety 4, no. 4: 171–181. 10.1177/2042098613489721. 25114779 PMC4125319

[fsn370479-bib-0008] Cho, H. , S. Jang , C. Won , et al. 2022. “Derhamnosylmaysin Inhibits Adipogenesis via Inhibiting Expression of PPARγ and C/EBPα in 3T3‐L1 Cells.” Molecules 27, no. 13: 4232. 10.3390/molecules27134232. 35807476 PMC9268393

[fsn370479-bib-0009] Fan, M. , Y. Choi , N. Wedamulla , et al. 2022. “Heat‐Killed *Enterococcus faecalis* EF‐2001 Attenuate Lipid Accumulation in Diet‐Induced Obese (DIO) Mice by Activating AMPK Signaling in Liver.” Food 11: 575. 10.3390/foods11040575. PMC8870772 35206052

[fsn370479-bib-0010] Ferré, P. , F. Phan , and F. Foufelle . 2021. “SREBP‐1c and Lipogenesis in the Liver: An update.” Biochemical Journal 478, no. 20: 3723–3739. 10.1042/bcj20210071. 34673919

[fsn370479-bib-0011] Filippatos, T. D. , C. S. Derdemezis , I. F. Gazi , E. S. Nakou , D. P. Mikhailidis , and M. S. Elisaf . 2008. “Orlistat‐Associated Adverse Effects and Drug Interactions: A Critical Review.” Drug Safety 31: 53–65. 10.2165/00002018-200831010-00005. 18095746

[fsn370479-bib-0012] Fischer, W. , A. Currais , Z. Liang , A. Pinto , and P. Maher . 2019. “Old Age‐Associated Phenotypic Screening for Alzheimer's Disease Drug Candidates Identifies Sterubin As a Potent Neuroprotective Compound From Yerba Santa.” Redox Biology 21: 101089. 10.1016/j.redox.2018.101089. 30594901 PMC6309122

[fsn370479-bib-0013] Fujiwara, N. , H. Nakagawa , K. Enooku , et al. 2018. “CPT2 Downregulation Adapts HCC to Lipid‐Rich Environment and Promotes Carcinogenesis via Acylcarnitine Accumulation in Obesity.” Gut 67, no. 8: 1493–1504. 10.1136/gutjnl-2017-315193. 29437870 PMC6039238

[fsn370479-bib-0014] Han, Y. , M. Lin , X. Wang , et al. 2015. “Basis of Aggravated Hepatic Lipid Metabolism by Chronic Stress in High‐Fat Diet‐Fed Rat.” Endocrine 48: 483–492. 10.1007/s12020-014-0307-x. 24895043

[fsn370479-bib-0015] Hofmann, J. , S. Fayez , M. Scheiner , et al. 2020. “Sterubin: Enantioresolution and Configurational Stability, Enantiomeric Purity in Nature, and Neuroprotective Activity In Vitro and In Vivo. Chemistry–A.” European Journal 26, no. 32: 7299–7308. 10.1002/chem.202001264. PMC7317536 32358806

[fsn370479-bib-0016] Hossain, M. , A. Dayem , J. Han , et al. 2016. “Molecular Mechanisms of the Anti‐Obesity and Anti‐Diabetic Properties of Flavonoids.” International Journal of Molecular Sciences 17, no. 4: 569. 10.3390/ijms17040569. 27092490 PMC4849025

[fsn370479-bib-0017] Im, S. , H. Kang , J. Kim , S. Kim , K. Kim , and S. Lee . 2024. “Narirutin‐Rich Celluclast Extract From Mandarin (*Citrus unshiu*) Peel Alleviates High‐Fat Diet‐Induced Obesity and Promotes Energy Metabolism in C57BL/6 Mice.” International Journal of Molecular Sciences 25, no. 8: 4475. 10.3390/ijms25084475. 38674060 PMC11049868

[fsn370479-bib-0018] Jayarathne, S. , I. Koboziev , O. Park , W. Oldewage‐Theron , C. Shen , and N. Moustaid‐Moussa . 2017. “Anti‐Inflammatory and Anti‐Obesity Properties of Food Bioactive Components: Effects on Adipose Tissue.” Preventive Nutrition and Food Science 22, no. 4: 251–262. 10.3746/pnf.2017.22.4.251. 29333376 PMC5758087

[fsn370479-bib-0019] Jung, J. , M. Lee , S. H. Park , et al. 2024. “Rose Petal Extract Ameliorates Obesity in High Fat Diet‐Induced Obese Mice.” Preventive Nutrition and Food Science 29, no. 2: 125–134. 10.3746/pnf.2024.29.2.125. 38974597 PMC11223920

[fsn370479-bib-0020] Kazemian, M. , M. Abad , M. R. Haeri , M. Ebrahimi , and R. Heidari . 2015. “Anti‐Diabetic Effect of *Capparis spinosa* L. Root Extract in Diabetic Rats.” Avicenna Journal of Phytomedicine 5, no. 4: 325–332. 26445712 PMC4587611

[fsn370479-bib-0021] Kazemipoor, M. , G. Cordell , M. Sarker , C. Radzi , M. Hajifaraji , and P. En Kiat . 2015. “Alternative Treatments for Weight Loss: Safety/Risks and Effectiveness of Anti‐Obesity Medicinal Plants.” International Journal of Food Properties 18, no. 9: 1942–1963. 10.1080/10942912.2014.933350.

[fsn370479-bib-0022] Lee, H. , S. Lim , J. Jung , et al. 2019. “ *Gynostemma pentaphyllum* Extract Ameliorates High‐Fat Diet‐Induced Obesity in C57BL/6N Mice by Upregulating SIRT1.” Nutrients 11, no. 10: 2475. 10.3390/nu11102475. 31618980 PMC6835433

[fsn370479-bib-0023] Lee, J. , J. M. Ellis , and M. J. Wolfgang . 2015. “Adipose Fatty Acid Oxidation Is Required for Thermogenesis and Potentiates Oxidative Stress‐Induced Inflammation.” Cell Reports 10, no. 2: 266–279. 10.1016/j.celrep.2014.12.023. 25578732 PMC4359063

[fsn370479-bib-0024] Leventhal‐Perek, S. , M. Shani , and Y. Schonmann . 2023. “Effectiveness and Persistence of Anti‐Obesity Medications (Liraglutide 3 mg, Lorcaserin, and Orlistat) in a Real‐World Primary Care Setting.” Family Practice 40, no. 5–6: 629–637. 10.1093/fampra/cmac141. 36477550

[fsn370479-bib-0025] Li, J. , H. Wu , Y. Liu , and L. Yang . 2020. “High Fat Diet Induced Obesity Model Using Four Strainsof Mice: Kunming, C57BL/6, BALB/c and ICR.” Experimental Animals 69, no. 3: 326–335. 10.1538/expanim.19-0148. 32188837 PMC7445062

[fsn370479-bib-0026] Li, X. , Z. Yao , X. Qi , et al. 2024. “Naringin Ameliorates Obesity via Stimulating Adipose Thermogenesis and Browning, and Modulating Gut Microbiota in Diet‐Induced Obese Mice.” Current Research in Food Science 8: 100683. 10.1016/j.crfs.2024.100683. 38313225 PMC10835601

[fsn370479-bib-0027] Lin, S. , X. Li , Q. Chen , et al. 2025. “Eriodictyol Regulates White Adipose Tissue Browning and Hepatic Lipid Metabolism in High Fat Diet‐Induced Obesity Mice via Activating AMPK/SIRT1 Pathway.” Journal of Ethnopharmacology 337: 118761. 10.1016/j.jep.2024.118761. 39216775

[fsn370479-bib-0028] Lin, X. , and H. Li . 2021. “Obesity: Epidemiology, Pathophysiology, and Therapeutics.” Frontiers in Endocrinology (Lausanne) 12: 706978. 10.3389/fendo.2021.706978. PMC8450866 34552557

[fsn370479-bib-0029] Mittendorfer, B. , R. E. Ostlund Jr. , B. W. Patterson , and S. Klein . 2001. “Orlistat Inhibits Dietary Cholesterol Absorption.” Obesity Research 9, no. 10: 599–604. 10.1038/oby.2001.79. 11595776

[fsn370479-bib-0030] Mödinger, Y. , C. Schön , M. Wilhelm , C. Pickel , and T. Grothe . 2021. “A Food Supplement With Antioxidative Santa Herba Extract Modulates Energy Metabolism and Contributes to Weight Management.” Journal of Medicinal Food 24, no. 11: 1235–1242. 10.1089/jmf.2021.0016. 34255555

[fsn370479-bib-0031] Mokdad‐Bzeouich, I. , N. Mustapha , A. Sassi , et al. 2016. “Investigation of Immunomodulatory and Anti‐Inflammatory Effects of Eriodictyol Through Its Cellular Anti‐Oxidant Activity.” Cell Stress and Chaperones 21, no. 5: 773–781. 10.1007/s12192-016-0702-8. 27250501 PMC5003794

[fsn370479-bib-0032] Müller, T. D. , M. Blüher , M. H. Tschöp , and R. D. DiMarchi . 2022. “Anti‐Obesity Drug Discovery: Advances and Challenges.” Nature Reviews Drug Discovery 21, no. 3: 201–223. 10.1038/s41573-021-00337-8. 34815532 PMC8609996

[fsn370479-bib-0033] Nani, A. , B. Murtaza , A. Sayed Khan , N. A. Khan , and A. Hichami . 2021. “Antioxidant and Anti‐Inflammatory Potential of Polyphenols Contained in Mediterranean Diet in Obesity: Molecular Mechanisms.” Molecules 26, no. 4: 985. 10.3390/molecules26040985. 33673390 PMC7918790

[fsn370479-bib-0034] NCD‐RisC . 2024. “Worldwide Trends in Underweight and Obesity From 1990 to 2022: A Pooled Analysis of 3663 Population‐Representative Studies With 222 Million Children, Adolescents, and Adults.” Lancet 403, no. 10431: 1027–1050. 10.1016/s0140-6736(23)02750-2. 38432237 PMC7615769

[fsn370479-bib-0035] Oda, N. , S. Imamura , T. Fujita , et al. 2008. “The Ratio of Leptin to Adiponectin Can Be Used As an Index of Insulin Resistance.” Metabolism 57, no. 2: 268–273. 10.1016/j.metabol.2007.09.011. 18191059

[fsn370479-bib-0036] Oliveira, A. K. S. , A. M. de Oliveira E Silva , R. O. Pereira , et al. 2022. “Anti‐Obesity Properties and Mechanism of Action of Flavonoids: A Review.” Critical Reviews in Food Science and Nutrition 62, no. 28: 7827–7848. 10.1080/10408398.2021.1919051. 33970708

[fsn370479-bib-0037] Oussaada, S. M. , K. A. Van Galen , M. I. Cooiman , et al. 2019. “The Pathogenesis of Obesity.” Metabolism 92: 26–36. 10.1016/j.metabol.2018.12.012. 30639246

[fsn370479-bib-0038] Pandeya, P. , R. Lamichhane , G. Lamichhane , et al. 2021. “18KHT01, a Potent Anti‐Obesity Polyherbal Formulation.” Frontiers in Pharmacology 12: 807081. 10.3389/fphar.2021.807081. 34975503 PMC8719591

[fsn370479-bib-0039] Pemmari, T. , M. Hämäläinen , R. Ryyti , R. Peltola , and E. Moilanen . 2022. “Cloudberry ( *Rubus chamaemorus* L.) Supplementation Attenuates the Development of Metabolic Inflammation in a High‐Fat Diet Mouse Model of Obesity.” Nutrients 14, no. 18: 3846. 10.3390/nu14183846. 36145221 PMC9503149

[fsn370479-bib-0040] Puljak, L. , V. Parameswara , S. Dolovcak , et al. 2008. “Evidence for AMPK‐Dependent Regulation of Exocytosis of Lipoproteins in a Model Liver Cell Line.” Experimental Cell Research 314, no. 10: 2100–2109. 10.1016/j.yexcr.2008.03.002. 18405894 PMC2699465

[fsn370479-bib-0041] Ramírez‐Moreno, E. , J. Arias‐Rico , R. C. Jiménez‐Sánchez , et al. 2022. “Role of Bioactive Compounds in Obesity: Metabolic Mechanism Focused on Inflammation.” Food 11, no. 9: 1232. 10.3390/foods11091232. PMC9101148 35563955

[fsn370479-bib-0042] Ren, R. , J. Gong , Y. Zhao , et al. 2018. “Sulfated Polysaccharide From *Enteromorpha prolifera* Suppresses SREBP‐1c and ACC Expression to Lower Serum Triglycerides in High‐Fat Diet‐Induced Hyperlipidaemic Rats.” Journal of Functional Foods 40: 722–728. 10.1016/j.jff.2017.12.010.

[fsn370479-bib-0043] Shaik Mohamed Sayed, U. , S. Moshawih , H. Goh , et al. 2023. “Natural Products As Novel Anti‐Obesity Agents: Insights Into Mechanisms of Action and Potential for Therapeutic Management.” Frontiers in Pharmacology 14: 1182937. 10.3389/fphar.2023.1182937. 37408757 PMC10318930

[fsn370479-bib-0044] Steinberg, G. R. , and D. Carling . 2019. “AMP‐Activated Protein Kinase: The Current Landscape for Drug Development.” Nature Reviews Drug Discovery 18, no. 7: 527–551. 10.1038/s41573-019-0019-2. 30867601

[fsn370479-bib-0045] Studentsova, V. , K. M. Mora , M. F. Glasner , M. R. Buckley , and A. E. Loiselle . 2018. “Obesity/Type II Diabetes Promotes Function‐Limiting Changes in Murine Tendons That Are Not Reversed by Restoring Normal Metabolic Function.” Scientific Reports 8, no. 1: 9218. 10.1038/s41598-018-27634-4. 29907811 PMC6003963

[fsn370479-bib-0046] Tak, Y. , and S. Lee . 2020. “Anti‐Obesity Drugs: Long‐Term Efficacy and Safety: An Updated Review.” World Journal of Men's Health 39, no. 2: 208–221. 10.5534/wjmh.200010. PMC7994651 32202085

[fsn370479-bib-0047] Toita, R. , T. Kawano , S. Fujita , M. Murata , and J.‐H. Kang . 2017. “Increased Hepatic Inflammation in a Normal‐Weight Mouse After Long‐Term High‐Fat Diet Feeding.” Journal of Toxicologic Pathology 31, no. 1: 43–47. 10.1293/tox.2017-0038. 29479139 PMC5820102

[fsn370479-bib-0048] Wali, J. A. , N. Jarzebska , D. Raubenheimer , S. J. Simpson , R. N. Rodionov , and J. F. O'Sullivan . 2020. “Cardio‐Metabolic Effects of High‐Fat Diets and Their Underlying Mechanisms—A Narrative Review.” Nutrients 12, no. 5: 1505. 10.3390/nu12051505. 32455838 PMC7284903

[fsn370479-bib-0049] Yan, J. , J. Bak , Y. Go , et al. 2023. “ *Scytosiphon lomentaria* Extract Ameliorates Obesity and Modulates Gut Microbiota in High‐Fat‐Diet‐Fed Mice.” Nutrients 15, no. 4: 815. 10.3390/nu15040815. 36839173 PMC9965426

[fsn370479-bib-0050] Yang, X. , and D. Shang . 2023. “The Role of Peroxisome Proliferator‐Activated Receptor γ in Lipid Metabolism and Inflammation in Atherosclerosis.” Cell Biology International 47, no. 9: 1469–1487. 10.1002/cbin.12065. 37369936

[fsn370479-bib-0051] Yang, Y. , H. Zhang , X. Li , T. Yang , and Q. Jiang . 2015. “Effects of PPARα/PGC‐1α on the Energy Metabolism Remodeling and Apoptosis in the Doxorubicin Induced Mice Cardiomyocytes In Vitro.” International Journal of Clinical and Experimental Pathology 8, no. 10: 12216–12224. 26722406 PMC4680351

[fsn370479-bib-0052] Zakaria, Z. , Z. A. Othman , J. Bagi Suleiman , N. A. C. Jalil , W. S. W. Ghazali , and M. Mohamed . 2021. “Protective and Therapeutic Effects of Orlistat on Metabolic Syndrome and Oxidative Stress in High‐Fat Diet‐Induced Metabolic Dysfunction‐Associated Fatty Liver Disease (MAFLD) in Rats: Role on Nrf2 Activation.” Veterinary Sciences 8, no. 11: 274. 10.3390/vetsci8110274. 34822647 PMC8622931

[fsn370479-bib-0053] Zhang, C. , J. Liu , X. He , et al. 2019. “Caulis Spatholobi Ameliorates Obesity Through Activating Brown Adipose Tissue and Modulating the Composition of Gut Microbiota.” International Journal of Molecular Sciences 20, no. 20: 5150. 10.3390/ijms20205150. 31627416 PMC6829277

